# Understanding Interfacial Reactions in Ti–Ni Diffusion Couple

**DOI:** 10.3390/ma16062267

**Published:** 2023-03-11

**Authors:** Amin Babaei-Dehkordi, Mansour Soltanieh, Mostafa Mirjalili, Mohammadreza Asherloo, Amir Mostafaei

**Affiliations:** 1 School of Metallurgy and Materials Engineering, Iran University of Science and Technology (IUST), Tehran 16846-13114, Iran; a.babaeidehkordi@gmail.com; 2 Department of Materials and Metallurgical Engineering, Faculty of Engineering, Ferdowsi University of Mashhad, Mashhad 91775-1111, Iran; 3 Department of Mechanical, Materials and Aerospace Engineering, Illinois Institute of Technology, 10 W 32nd Street, Chicago, IL 60616, USA

**Keywords:** Ti–Ni system, intermetallic compounds, integrated diffusion coefficient, scanning electron microscopy

## Abstract

The diffusion phenomenon in the Ti–Ni binary system was investigated at a temperature of 1173 K. Microstructure and texture analysis revealed the formation of three stable intermetallic compounds, namely Ti_2_Ni, TiNi, and TiNi_3_, as well as two metastable intermetallic compounds, including Ti_3_Ni_4_ and Ti_2_Ni_3_, at the interfacial diffusion zone. The nucleation surface energy increase was analytically estimated, and marker experiments were conducted using thoria particles, both of which showed that Ti_2_Ni was the first compound to form at the Ti–Ni diffusion interface. At a temperature of 1173 K, using the Wagner method, the integrated diffusion coefficients for the Ti_2_Ni, TiNi, and TiNi_3_ phases were calculated to be 3.53 × 10^−12^, 18.1 × 10^−15^, and 6.2 × 10^−15^ m^2^/s, for, respectively.

## 1. Introduction

Ti–Ni alloys, known for their exceptional properties such as shape memory effect, corrosion resistance, shock absorption, superelasticity, and biocompatibility, have found applications in diverse fields ranging from aerospace, automotive, and oil to biomedical sectors [1,2,3,4]. Given their importance, ongoing research in the field of the Ti–Ni binary system is focused on exploring its potential applications [5,6,7,8]. Depending on the heat treatment conditions, the Ti–Ni binary system can produce three stable intermetallic compounds, namely Ti_2_Ni, TiNi, and TiNi_3_, as well as two metastable intermetallic compounds, Ti_3_Ni_4_ and Ti_2_Ni_3_ [9]. However, limited attention has been given to the diffusion coefficients of alloying elements, which significantly affect the kinetics of intermetallic compound formation [1,10,11,12,13,14,15].

In the Ti–Ni binary system, TiNi is the most important compound, and its formation is influenced by the two other stable phases, Ti_2_Ni and TiNi_3_. Therefore, understanding the sequence and formation mechanism of TiNi is crucial. A diffusion couple study is a practical approach to investigate diffusion phenomena in solid-state conditions. The formation of different intermetallic compounds in the Ti–Ni binary system is based on diffusional transformation, including the metastable phases of Ti_3_Ni_4_ → Ti_2_Ni_3_ → TiNi_3_ (stable), where temperature and holding duration are critical factors that determine the kinetics of this process [16].

The presence of metastable phases such as Ti_3_Ni_4_ and Ti_2_Ni_3_ in Ti–Ni alloys can affect their shape memory behavior [17,18,19,20]. However, in earlier studies investigating the formation of different intermetallic compounds in the Ti–Ni binary system using diffusion coupling, the formation of these metastable phases was disregarded [21,22,23,24,25]. The diffusion coefficient of elements plays a critical role in the kinetics of diffusion during the solid-state formation of intermetallic compounds, influencing the diffusion mechanism and the sequence of intermetallic compound formation. The kinetics of growth are governed by volume diffusion and interdiffusion coefficients, which can effectively compare growth rates between different intermetallic phases [26].

Previous studies have overlooked the formation of Ti_3_Ni_4_ and Ti_2_Ni_3_ metastable intermetallic compounds in the Ti–Ni binary system. Therefore, the primary objective of this study is to investigate the formation of these metastable compounds using the integrated diffusion coefficient of elements. In this research, both experimental and analytical analyses were conducted to gain a comprehensive understanding of the formation of various intermetallic compounds in the Ti–Ni binary system.

## 2. Materials and Methods

To investigate the interfacial reactions and formation sequence of various phases and intermetallic compounds at the Ti–Ni diffusion couple interface, high purity commercial Ni (1 mm thick) and Ti (2 mm thick) sheets were purchased. A Ti sheet was placed between two Ni plates to form the diffusion couple. The sheets were sliced to dimensions of 15 mm × 6 mm and their surfaces were ground using SiC sandpaper up to 2500 grit. To minimize the presence of any oxide film or potential contaminations at the Ni–Ti–Ni interfaces, the metal sheets were ultrasonically etched in a mixed acidic solution consisting of 10% HCl, 67% HNO3, and 23% deionized water. The sheets were then rinsed in acetone and dried. To ensure maximum surface contact at the Ni–Ti–Ni interfaces, a steel fixture was used. The annealing treatment was conducted in a tube furnace at a temperature of 1173 ± 5 K under an Ar atmosphere. The quartz tube was vacuumed and then backfilled with ultra-high purity Ar gas (99.999% purity) to minimize oxidation.

The microstructure at the interface of the Ti–Ni binary system was studied using a standard metallographic procedure, which included a final polishing step of 0.05 µm colloidal silica. The morphology, composition, and thickness of the formed intermetallic compounds at the Ti–Ni interface were analyzed using a scanning electron microscope (SEM, TESCAN VEGA//XMU) equipped with an energy-dispersive spectrometer (EDS). The morphological structure of the Ti sheet was observed using an optical microscope (MEIJI TECHNO, MT7350, Japan). Texture and phase analyses were performed using a JEOL JSM 5900-LV SEM equipped with an Oxford Instruments Nordlys Nano electron backscatter diffraction (EBSD) detector. The EBSD imaging was conducted at an accelerating voltage of 20 keV, and the data were analyzed using Channel 5-HKL software.

## 3. Results and Discussion

**Figure 1:**
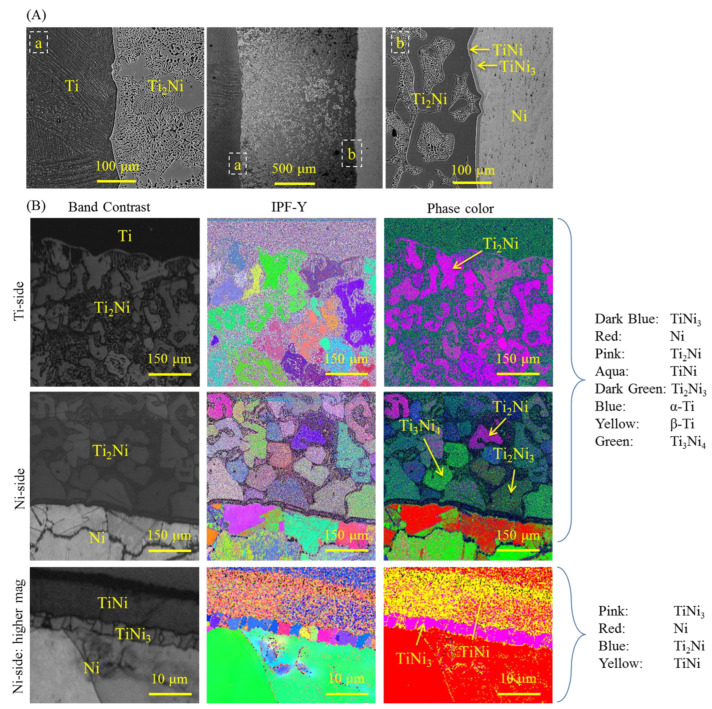
(**A**) SEM micrographs from the Ti–Ni diffusion couple annealed at 1173 K for 13 h (etched by Kroll’s reagent). (**B**) Electron backscattered diffraction from the Ti–Ni interface.

Figure 1a shows SEM images of the Ti–Ni interface, which appears fairly straight, suggesting uniform pressure on the flat surfaces. Elemental analysis revealed that the layer adjacent to the Ti sheet had a composition of 68.9 at% Ti and 31.1 at% Ni, indicating the dominant formation of a Ti_2_Ni intermetallic phase after annealing at 1173 K for 13 h. Two narrow intermetallic layers were also detected near the Ni side, with the TiNi layer composed of 50.6 at% Ti and 49.4 at% Ni, and the TiNi_3_ layer adjacent to the Ni sheet containing an elemental fraction of 26.3 at% Ti and 73.7 at% Ni. These observations are consistent with the Ti–Ni binary diagram and previous studies [21,22,23,25,27,28,29]. The Ti_2_Ni layer was thicker than the TiNi and TiNi_3_ layers and had an island microstructure. To analyze the crystal structure of the three intermetallic compounds and investigate the nucleation mechanism and growth behavior at the Ti–Ni interface, EBSD analysis was performed. The results in Figure 1b show the presence of two additional intermetallic compounds, Ti_3_Ni_4_ and Ti_2_Ni_3_, at the Ti–Ni interface in addition to the Ti_2_Ni, TiNi, and TiNi_3_ intermetallic compounds. Ti_3_Ni_4_ and Ti_2_Ni_3_ are identified as metastable phases in the Ti–Ni phase diagram [30].

The grain size distribution in the diffusion layers was observed in the EBSD results (Figure 1b) taken from both the Ti and Ni sides. Fine-grained Ti_2_Ni was formed close to the Ti side, while coarse mixed grains were formed on the Ni side. The unindexed areas close to the Ti side were attributed to the fine-grained structure of the Ti alloy. To elucidate the formation mechanism, it is crucial to identify the dominant diffusing element, determine the diffusion coefficients of the elements, and investigate the first intermetallic layer formed at the Ti–Ni binary system. The sequence of intermetallic compound formation at the Ti–Ni interface can be determined by calculating the Gibbs free energies of formation for the three compounds as a function of temperature [25].

The formation of intermetallic compounds in the Ti–Ni binary system is a complex process that is governed by kinetics. Although the calculation of Gibbs free energy is important, it alone is not sufficient to determine the order of intermetallic formation. The formation process involves a series of events such as the diffusion of Ni and Ti, formation of saturated primary solid solutions, nucleation, and growth of an equilibrium phase in a sequential manner. When all the necessary conditions are met, a new phase will be formed at the interface of Ti–Ni.

In the Ti–Ni binary system, the interdiffusion phenomenon leads to the formation of saturated solid solutions of Ti (Ni) and Ni (Ti) on either side. The lower solubility limit of Ni in Ti at the annealing temperature resulted in the formation of the Ti (Ni) solid solution and nucleation of the Ti_2_Ni phase. According to nucleation theory, a compound with the lowest interfacial energy will nucleate more easily than other compounds at the diffusion zone [31].

The determination of the increasing interfacial energy involves several factors, including the interfacial energy of the initial *A*/*B* interface (*γ_A-B_*, *A* or *B* can be either Ti or Ni), the surface energy of the *AB* phase (*γ_AB_*, where *AB* can be Ti_2_Ni, Ti_3_Ni_4_, Ti_2_Ni_3_, TiNi, or TiNi_3_), the surface energy of the initial substances (*γ_i_*, where *i* = Ti or Ni) and the interfacial energy of the new interface *AB*/*A* or *AB*/*B* (*γ_A-AB_* or *γ_B-AB_*). These terms can be calculated using the following equations [32,33]:

(1) $$\gamma_{A-B\;}=\frac{1}{6}\;\left(\gamma_{A}+\gamma_{B}\;\right)+\frac{\Delta H_{A\;in\;B}^{interface}}{C_{o}V_{A}^{2/3}}$$

(2) $$\gamma_{AB\;}=C_{A}^{S}\gamma_{A}+C_{B}^{S}\gamma_{B}-C_{A}^{S}C_{B}^{S}\;\frac{\Delta H_{A\;in\;B}^{interface}}{C_{0}V_{A}^{2/3}}$$

(3) $$\gamma_{A-AB\;}=\frac{1}{6}\;\left(\gamma_{A}+\gamma_{AB}\;\right)+\frac{C_{B}^{S}\Delta H_{A\;in\;B}^{interface}}{C_{o}V_{A}^{2/3}}$$

(4) $$\Delta H_{A\;in\;B}^{interface}=\frac{2PV_{A}^{2/3}}{n_{A}^{-1/3}+n_{B}^{-1/3}}[-{\left({ᶲ}_{A}-{ᶲ}_{B}\right)}^{2}+\frac{Q{\left(n_{A}^{\frac{1}{3}}-n_{B}^{\frac{1}{3}}\right)}^{2}}{P}-\frac{R}{P}$$

(5) $$C_{A}^{S}=C_{A}V_{A}^{\frac{2}{3}}/\left(C_{A}V_{A}^{\frac{2}{3}}+C_{B}V_{B}^{\frac{2}{3}}\right)$$

(6) $$C_{B}^{S}=C_{B}V_{B}^{\frac{2}{3}}/\left(C_{A}V_{A}^{\frac{2}{3}}+C_{B}V_{B}^{\frac{2}{3}}\right)$$

where $\Delta H_{A\;in\;B}^{interface}$ is the enthalpy change upon the solution of 1 mole of *A* in *B*, *V_A_* is the molar volume of Ti or Ni atoms (*V_Ti_* = 10.6 cm^3^/mol, *V_Ni_* = 6.54 cm^3^/mol) [33], *C_o_* is a constant, taken as 4.5 × 10^8^ [31], *C_A_* and *C_B_* are the concentrations of *A* and *B* atoms, respectively, *γ_A_* is the surface energy (*γ_Ni_* = 2000 mJ/m^2^, *γ_Ti_* = 2051 mJ/m^2^), $n_{i}$ is the electron density ($n_{Ti}^{\frac{1}{3}}$ = 1.47, $n_{Ni}^{\frac{1}{3}}$ = 1.75). $\frac{Q}{P},\;\frac{R}{P}\;\mathrm{and}\;P$ are constant values and are equal to 9.4, 1.9, and 12.35, respectively [33]. $C_{A}^{S}$ and $C_{B\;}^{S}$ are the surface fraction of A and B atoms [31]. Based on Equations (1)–(6), the *γ*_Ti-Ni_ is −106.37 mJ/m^2^ and the increasing interface energies of Ti_2_Ni, Ti_3_Ni_4_, Ti_2_Ni_3,_ TiNi, and TiNi_3_ compounds is calculated and listed in Table 1. The formation of Ti_2_Ni, Ti_3_Ni_4_, Ti_2_Ni_3_, TiNi, and TiNi_3_ compounds is associated with a 647.5, 891, 686.1, 674.1, and a 692.2 mJ/m^2^ increase in interface energy, respectively. Based on the results, it can be inferred that the formation of Ti_2_Ni has the lowest interface energy, suggesting that Ti_2_Ni nucleates first in the Ti–Ni diffusion couple.

**Figure 2:**
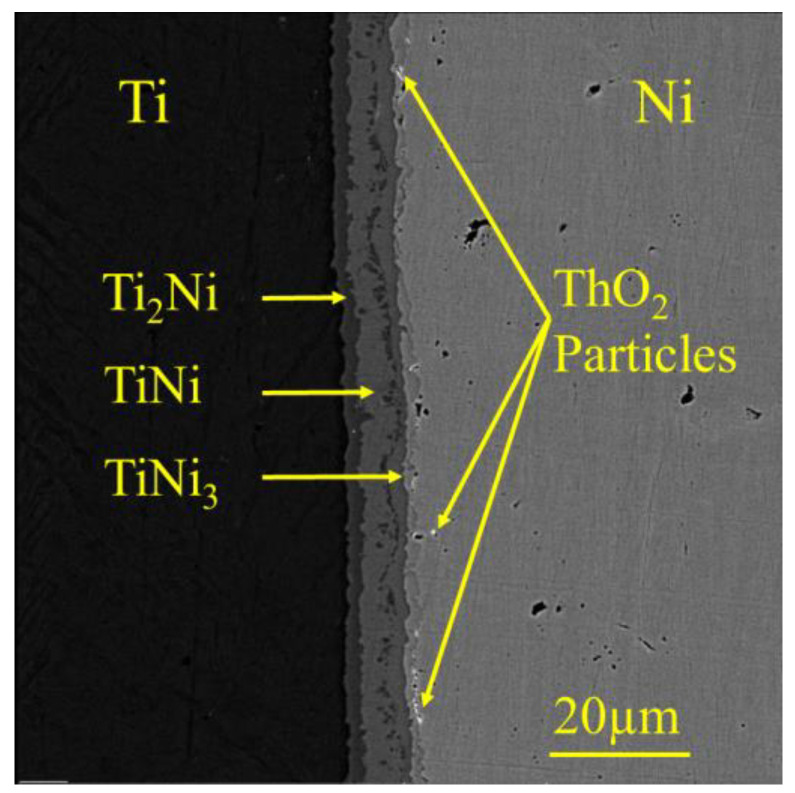
SEM micrograph at the interface of the Ti–Ni diffusion couple. To study the diffusion behavior, ThO_2_ particles were placed at the Ti–Ni interface. The sample was annealed at 1173 K for 5 h.

In this study, the marker test was used to determine the predominant diffusing element by placing ThO_2_ particles at the Ti–Ni interface. As ThO_2_ particles act as a marker for the true position of the Kirkendall plane [34], their location revealed the diffusing element. Figure 2 shows the location of the ThO_2_ particles at the Ni/Ni_3_Ti interface. According to EDS results, the first layer adjacent to the Ti side contained 68.5 at% Ti and 31.5 at% Ni, while the next layer contained 51.3 at% Ti and 49.7 at% Ni. The layer adjacent to the Ni side contained 26.3 at% Ti and 73.7 at% Ni, indicating the formation of Ti_2_Ni, TiNi, and TiNi_3_, respectively.

In the Ti–Ni binary system, the ratio of intrinsic diffusivities can be determined at the location of the Kirkendall marker plane using the following Equation (7) [35,36]:

(7) $$\frac{V_{Ti}D_{Ni}}{V_{Ni}D_{Ti}}=\frac{D_{Ni}^{*}}{D_{Ti}^{*}}=\frac{N_{Ni}^{+}\int_{x^{-\infty }}^{x_{k}}\left(N_{Ni}-N_{Ni}^{-}\right)dx-N_{Ni}^{-}\int_{x^{k}}^{x_{+\infty }}\left(N_{Ni}^{+}-N_{Ni}\right)dx}{-N_{Ti}^{+}\int_{x^{-\infty }}^{x_{k}}\left(N_{Ni}-N_{Ni}^{-}\right)dx\mp N_{Ti}^{-}\int_{x^{k}}^{x_{+\infty }}\left(N_{Ni}^{+}-N_{Ni}\right)dx}$$

where *D_i_* is the intrinsic diffusion coefficient, $D_{i}^{*}$ is the tracer diffusion coefficient, *V_i_* is the partial molar volume of element *i*, $x_{k}$ is the Kirkendall marker plane location, and $x^{-\infty }$ and $x^{+\infty }$ correspond to the unaffected ends of the diffusion couple [37]. Due to the position of the marker location at the Ni/TiNi_3_ interface, it can be concluded that $\frac{D_{Ni}^{*}}{D_{Ti}^{*}}=\infty$, meaning that Ni was the dominant diffusing element in the Ni–Ti diffusion couple. These observations are consistent with the analytical calculations of the increasing interface energy of the intermetallic phases in the Ti–Ni interface, as described by Equations (1)–(6).

**Figure 3:**
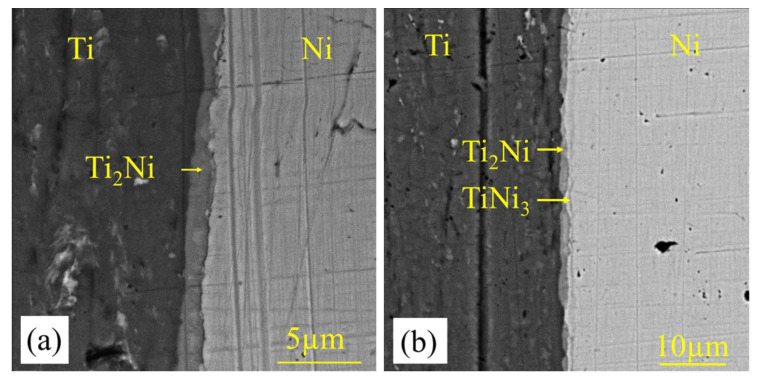
SEM micrographs at the interface of the Ti–Ni diffusion couple annealed at 900 °C for (**a**) 1 min and (**b**) 5 min.

Based on the EDS results, the layer formed between the Ti and Ni sheets in Figure 3a contains 66.4 at% Ti and 33.6 at% Ni, which is most likely due to the formation of Ti_2_Ni. Similarly, the layer formed on the Ti side in Figure 3b contains 67 at% Ti and 33 at% Ni, while the next layer contains 23 at% Ti and 77 at% Ni, indicating the formation of Ti_2_Ni and TiNi_3_, respectively. These results suggest that the Ti_2_Ni intermetallic compound is the first phase formed at the interface of the Ti–Ni diffusion couple. These observations are supported by numerical calculations and marker experiments.

After formation of the Ti_2_Ni layer, two new interfaces, namely Ti/Ti_2_Ni and Ti_2_Ni/Ni, are formed. These interfaces differ from the original Ti/Ni interface and cannot be described by Equations (1)–(6). Previous studies have reported the formation of three main intermetallic compounds, including Ti_2_Ni, TiNi, and TiNi_3_ [21,22,23], while the formation of metastable phases such as Ti_3_Ni_4_ and Ti_2_Ni_3_ has been overlooked. Due to the higher diffusion rate of Ni toward Ti [24], a saturated solid solution of Ti (Ni) is formed, and a fine Ti_2_Ni phase is nucleated near the Ti side, as shown in Figure 1a. With prolonged annealing treatment, grain coarsening occurs in the Ti_2_Ni layer close to the Ti side. The Ni continues to diffuse through this layer, and the metastable phase Ti_3_Ni_4_ is formed according to the following equation:

3/2Ti_2_Ni + 5/2Ni → Ti_3_Ni_4_ (8)

Excessive diffusion of Ni can result in the formation of another metastable phase including Ti_2_Ni_3_, according to the following equation:

2/3Ti_3_Ni_4_ + 1/3Ni → Ti_2_Ni_3_ (9)

Subsequently, as shown in Figure 3b, the stable TiNi_3_ intermetallic compound was formed on the Ni side according to the following equation:

2Ti_2_Ni_3_ + 6Ni → 4TiNi_3_ (10)

Finally, a stable TiNi phase was formed in the interface of the Ti_2_Ni and TiNi_3_ layers, as shown in Figure 3b, according to the following equation:

2Ti_2_Ni + TiNi_3_ → 5TiNi (11)

The subsequent formation and growth of the intermetallic layers are affected by the diffusion of Ti and Ni elements through the formed layers. After the formation of a continuous TiNi layer at the Ti_2_Ni/TiNi_3_ interface, the Ti_2_Ni/TiNi_3_ interface disappeared, and new interfaces were formed, including TiNi/TiNi_3_ and Ti_2_Ni/TiNi. Therefore, the formation and growth of the TiNi layer can be explained by two other reactions instead of Equation (11). To determine the subsequent growth of the intermetallic layers, it is necessary to identify the dominant diffusing element.

Based on the marker experiment, it can be inferred that Ni is the predominant diffusing element in the Ti–Ni binary system due to its lower melting temperature and smaller atomic radius (1728 K, 163 pm) compared with Ti (1941 K, 187 pm) [38]. As a result, faster diffusion of the Ni element through the formed layers at the Ti/Ti_2_Ni interface can potentially form a fine-grained structure of Ti_2_Ni. As per Equation (12), a portion of the diffused Ni reacts with Ti_2_Ni at the Ti_2_Ni/TiNi interface, leading to the formation of the TiNi layer. The dissolution of Ti_2_Ni grains and their conversion to TiNi grains lead to the formation of a dendrite-like structure at the Ti_2_Ni/TiNi interface in accordance with previous studies [21,36].

1/2Ti_2_Ni + 1/2Ni → TiNi (12)

At the TiNi/TiNi_3_ interface, part of the diffused Ti through the Ti_2_Ni and TiNi layers reacts with the TiNi_3_ intermetallic compound, forming and growing the TiNi layer, as given in Equation (13).

1/3 TiNi_3_ + 2/3Ti → TiNi (13)

It should be noted that the formation rate of the TiNi_3_ layer is expected to be lower than that of the Ti_2_Ni layer due to the higher diffusivity of Ni. Moreover, the accumulation of vacancies at the TiNi_3_/Ni interface decreases the diffusion of Ni over a prolonged annealing time, and the TiNi_3_ layer acts as an Ni source. Finally, the lower formation of the TiNi_3_ layer compared with the consumption of this layer results in the consumption of the TiNi_3_ layer, which is in agreement with other results [28].

The diffusion coefficient of elements is a key factor to determining the sequence formation of phases and growth mechanism in the Ti–Ni diffusion couples. The Wagner equation [39,40] (Equation (14)) can be used to calculate the diffusion coefficient in a multi-component structure:

(14) $$\begin{array}{l} {\tilde{D}}_{int}^{\beta }=\frac{\left(N_{i}^{\beta }-N_{i}^{-}\right)\left(N_{i}^{+}-N_{i}^{\beta }\right)}{N_{i}^{+}-N_{i}^{-}}\frac{\Delta x_{\beta }^{2}}{2t}+\\ \;\;\;\;\;\;\;\;\;\;\;\;\;\;\;\;\;\;\;\;\;\;\frac{\Delta x_{\beta }}{2t}[\frac{\left(N_{i}^{+}-N_{i}^{\beta }\right)\sum_{v=2}^{v=\beta -1}\frac{V_{m}^{\beta }}{V_{m}^{v}}\left(N_{i}^{v}-N_{i}^{-}\right)\Delta x_{v}+\left(N_{i}^{\beta }-N_{i}^{-}\right)\sum_{v=\beta +1}^{v=n-1}\frac{V_{m}^{\beta }}{V_{m}^{v}}\left(N_{i}^{+}-N_{i}^{v}\right)\Delta x_{v}}{N_{i}^{+}-N_{i}^{-}} \end{array}$$

where ${\tilde{D}}_{int}^{\beta }$ (m^2^/s) is the integrated diffusion coefficient, $N_{i}^{-}$ and $N_{i}^{+}$ are the mole fractions of component *i* in the unreacted left- and right-hand sides of the ends of the couple, respectively. $N_{i}^{\beta }$ and $N_{i}^{v}$ are the mole fractions of component *i* in the phase of interest *β* and *ν*, respectively, $V_{m}^{v}$ and $\Delta x_{v}$ are the molar volume and the layer thickness of the *v* phase, and *t* is the annealing time. The data used to calculate the integrated diffusion coefficients of the Ti_2_Ni, TiNi, and TiNi_3_ phases formed in the Ti–Ni diffusion couple after annealing at 1173 K for 13 h are represented in Table 2. Based on Equation (14), the integrated diffusion coefficients for the Ti_2_Ni, TiNi, and TiNi_3_ phases were 3.53 × 10^−12^, 18.1 × 10^−15^, and 6.2 × 10^−15^ m^2^/s, respectively.

The integrated diffusion coefficient calculated in this study is not consistent with the results presented in [40], which showed that the integrated diffusion coefficient is higher in the Ti_2_Ni layer. Grain boundaries play an important role in accelerating the diffusion phenomena, as they act as fast diffusion paths and promote formation of intermetallic layers [26]. The activation energy for grain boundary diffusion is about half that for lattice diffusion [41], and the grain boundary diffusion coefficient (*D_gb_*) is much greater than the diffusion coefficient in bulk (*D_l_*). Diffusion is faster in fine-grained solids; thus, the mass transport process is affected by the grain size of the polycrystalline material [42].

**Figure 4:**
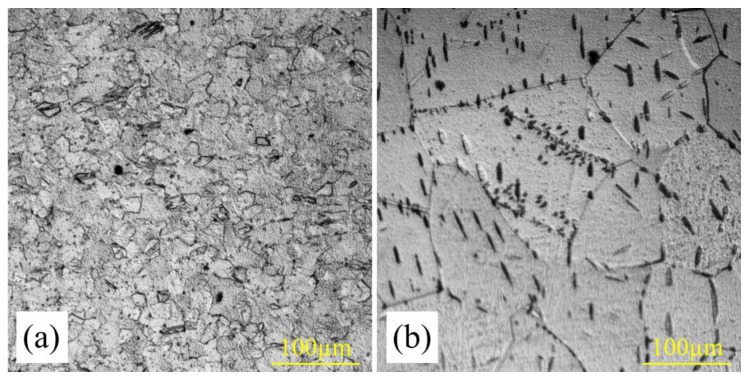
Optical micrograph of the cross-section of Ti sheet (**a**) as received without any pre-annealing process and (**b**) annealed at 1033 K for 150 min.

In this research, a Ti sheet was used as received without any pre-annealing process, unlike our earlier study [40], in which an annealed titanium sheet (at 1023 K for 3 h holding time) was used. As shown in Figure 4, the higher integrated diffusion coefficient in Ti_2_Ni could be attributed to the finer structure of the titanium used. Moreover, the Ti has a bcc crystal structure at 1173 K with a lower packing factor (PF) compared with the hcp crystal structure at lower temperatures (<1155 K). Therefore, it is expected that the Ti_2_Ni layer forms faster than the other intermetallic layers, and the integrated diffusion coefficient is greater in this layer than the other layers, in agreement with the calculation results. Using finer-grained Ti is expected to result in the formation of intermetallic layers at a shorter annealing time. Hence, the difference in the diffusion coefficient value could be related to the difference in the grain size of the primary sheets.

## 4. Conclusions

The diffusion phenomenon in the Ti–Ni binary system was investigated at 1173 K. It was found that:

1. Three intermetallic compounds in the sequence of Ti_2_Ni, TiNi, and TiNi_3_ and two metastable intermetallic compounds including Ti_3_Ni_4_ and Ti_2_Ni_3_ were formed at the Ti–Ni interface.
2. The marker technique and calculating the ratio of intrinsic diffusivities indicated that Ni is the dominant diffusing element.
3. The integrated diffusion coefficients, calculated using the Wagner method, were 3.53 × 10^−12^, 18.1 × 10^−15^, and 6.2 × 10^−15^ m^2^/s for Ti_2_Ni, TiNi, and TiNi_3_ at 1173 K, respectively.
4. Annealing of the titanium sheet resulted in grain growth, which reduced the contribution of grain boundaries to the overall diffusion and resulted in the reduction. As a result, the integrated diffusion coefficient in the Ti_2_Ni layer decreased.

## Figures and Tables

**Table 1:** The increasing interface energies of different first formation phases.

Phases	Surface Energy *γ* (AB) (mJ/m^2^)	Interface Energy *γ* (Ti-AB) (mJ/m^2^)	Interface Energy *γ* (Ni-AB) (mJ/m^2^)	Increasing Interface Energy (mJ/m^2^)
Ti_3_Ni_4_	2222.4	328.8	455.7	891.0
Ti_2_Ni_3_	2220.6	305.0	274.6	686.1
TiNi_3_	2185.1	169.6	416.3	692.2
Ti_2_Ni	2191.2	498.0	42.8	647.5
TiNi	2220.9	382.5	185.4	674.1

**Table 2:** Experimental data after annealing of Ti–Ni diffusion couple for 13 h at 1173 K.

Phase, j	Ti	Ti_2_Ni	TiNi	TiNi_3_	Ni
Thickness, μm	-	1391	11	6	-
Ni Mole fraction, N_Ni_	0.03	0.32	0.49	0.74	1
Ti Mole fraction, N_Ti_	0.97	0.68	0.51	0.26	0
Molar volume (V_j_, cm^3^)	-	9	8.2	7	-

## Data Availability

Data will be made available on request.
